# Eliminating Cholera Incidence and Mortality: Unfulfilled Tasks

**DOI:** 10.3390/tropicalmed7050069

**Published:** 2022-05-09

**Authors:** David Nalin

**Affiliations:** Center for Immunology and Microbial Diseases, Albany Medical College, Albany, NY 12208, USA; nalindavid@gmail.com

Impressive advances have been made in new cholera vaccine development and vaccination control strategies. Possible future goals in this field could extend these advances by developing vaccines with higher efficacy and longer duration of protection, particularly in young children and individuals from non-endemic areas. The identification of more vibrio antigens may lead to confirmation of protective immune responses as surrogates of protection, a need arising from evidence that the traditionally monitored vibriocidal response, while paralleling evidence of protection, is not the protective mechanism. Such further developments could overcome current limitations, including the occurrence of cholera outbreaks in war-torn areas in which short-term vaccination programs often prove impracticable.

The advances in understanding of cholera immunology, bioecology, vaccine innovation and therapy presented in this series of articles have led to ambitious goals for controlling the incidence and the mortality of cholera, which persists in affected areas. The discussion would not be complete without noting areas not included or given priority in the current goals, but which may prove to be of value for achieving them. 

First, there is too much talk and too little pressure brought to bear on the need for action to provide safe chlorinated drinking water and sanitary waste disposal to unserved areas. More effort is required to reframe national priorities so that adequate funds for these essential elements of modern public health are provided in both urban and rural environments, along with the educational and motivational components to ensure their effective usage. International standards and regulations governing urban development in the age of global urbanization [1] are essential if the Global Task Force on Cholera Control’s goal of ending cholera by 2030 is to have any chance of succeeding.

Second, insufficient attention has been given to the etiologies and prevention, nutritional and otherwise, of tropical hypochlorhydria [2], which is widespread in the developing nations and renders their populations highly susceptible to cholera and other pathogens sensitive to gastric acid. Based on human volunteer studies [3], which established that even enormous numbers of *V. cholerae* fail to cause disease in normochlorhydric subjects, it is likely that elimination of tropical hypochlorhydria would greatly reduce cholera incidence in affected areas and potentially make vaccines significantly more protective.

Third, far too little research funding has been directed at discovering safe and effective anti-cholera medicines capable of quickly stopping cholera diarrhea. No mass screening of compounds likely to have such efficacy has been undertaken despite an abundance of potential candidates. Recent advances in cholera pathophysiology, such as confirmation of the role of VIP in human cholera [4], suggest a number of potential high-value targets which merit inclusion in such a screening program in animal models leading to clinical trials.

Finally, the continued high cholera case-fatality rates despite established highly effective and widely available treatment modalities demand renewed focus on the gaps preventing therapy from reaching patients.
